# COVID-19 and acute pulmonary embolism: what should be considered to indicate a computed tomography pulmonary angiography scan?

**DOI:** 10.1590/0037-8682-0267-2020

**Published:** 2020-06-01

**Authors:** Bruno Lima Moreira, Pablo Rydz Pinheiro Santana, Gláucia Zanetti, Edson Marchiori

**Affiliations:** 1 BP Medicina Diagnóstica - A Beneficência Portuguesa de São Paulo, São Paulo, SP, Brasil.; 2 Grupo Fleury, São Paulo, SP, Brasil.; 3 Universidade Federal do Rio de Janeiro, Rio de Janeiro, RJ, Brasil.

**Keywords:** COVID-19 (coronavirus disease 2019), SARS-CoV-2 (severe acute respiratory syndrome coronavirus 2), Computed tomography angiography

## Abstract

The full spectrum of COVID-19 is still emerging, although several studies have highlighted that patients infected with the novel coronavirus can potentially develop a hypercoagulable state. However, several aspects related to the incidence and pathophysiology of the association between COVID-19 and pulmonary embolism are not well established. Here, we present a case of a patient with COVID-19 who developed acute pulmonary embolism. Clinical and laboratory data and findings of non-enhanced CT indicate possibility of acute pulmonary embolism, and support the decision to proceed with computed tomography pulmonary angiography that can objectively identify filling defects in pulmonary arterial branches.

## INTRODUCTION

Coronavirus disease 2019 (COVID-19) is caused by a betacoronavirus named severe acute respiratory syndrome coronavirus 2 (SARS-CoV-2). Since its outbreak in Wuhan, China, in December 2019, the novel coronavirus has spread worldwide. On March 11, 2020, the World Health Organization declared the COVID-19 as a pandemic^1^.

Most of the patients with COVID-19 have mild disease symptoms^2^. However, few patients can develop acute respiratory distress syndrome (ARDS) and other complications, including thrombotic phenomena in macro- and micro-circulation^2^ ^-^ ^5^.Coagulopathy is commonly observed in ARDS/sepsis and can predict outcomes in severe/critical COVID-19 cases^2^ ^-^ ^5^ ^,^ ^3^ ^-^ ^6^. Abnormal coagulation parameters (elevated levels of D-dimer and fibrin degradation products) significantly correlate with mortality in patients with COVID-19^6^. Moreover, the SARS-CoV-2 may induce a cytokine storm along with activation of the coagulation cascade that leads to the thrombotic phenomena^5^ ^,^ ^7^.

Further, pulmonary embolism (PE) in patients with COVID-19 has received increasing attention in the medical community^1^ ^,^ ^3^ ^,^ ^8^ ^,^ ^9^. These patients are prone to an increased risk of venous thromboembolism^1^ ^,^ ^8^.

## CASE REPORT

**FIGURE 1:**
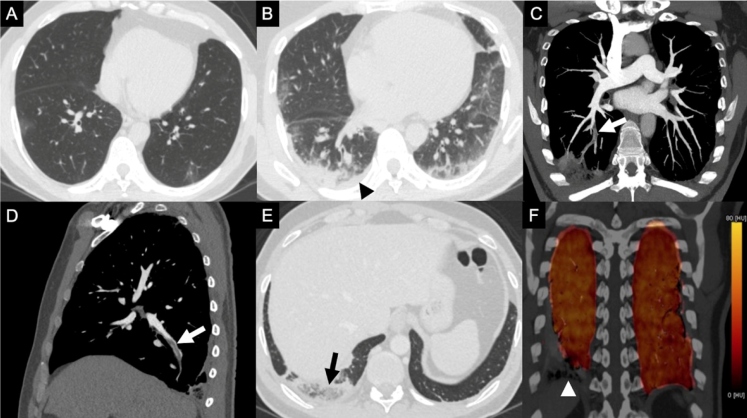
Initial non-enhanced axial chest CT image **(A)** shows few scattered peripheral ground-glass opacities in both the lungs. Non-enhanced axial chest CT image obtained 14 days later **(B)** indicates an increased extent of the ground-glass opacities and development of consolidation, especially in the posterior periphery of the right lower lobe. A new right-sided laminar pleural effusion (black arrowhead) can be observed. Note the difference in heart volume between images A and B (the heart volume is greater in image B, although within normal limits). CT pulmonary angiography images in the coronal-oblique plane with maximum intensity projection **(C)** and sagittal plane **(D)** show acute emboli (white arrows) in the sub-segmental arterial branches of the posterior basal segment of the right lower lobe. CT pulmonary angiography image (lung window) in the axial plane (E) depicts peripheral opacity with features compatible with pulmonary infarction (reversed halo sign with internal reticulation and low-attenuation areas, black arrow) in the posterior basal segment of the right lower lobe. Dual-energy CT color-coded iodine “perfusion” map in the coronal plane (F) suggesting focal iodine defect (white arrowhead) only in the area of pulmonary infarction in the right lower lobe.

Here, we present the case of a 52-year-old male patient who visited our emergency department with a 5-day history of fever, myalgia, abdominal pain, headache, cough, and dyspnea. The patient reported asthma, but no recent attack. He denied other comorbidities, smoking, or continued medication use. On admission, the patient’s vital signs were normal, with peripheral oxygen saturation (SpO_2_) >95%. The white blood cell (WBC) count and C-reactive protein level showed no significant abnormalities. Rapid influenza diagnostic test was negative. The patient underwent a non-enhanced chest computed tomography (CT) scan that revealed few scattered ground-glass opacities in both the lungs, with a predominantly peripheral distribution (Figure 1A). Real-time reverse-transcription polymerase chain reaction (rRT-PCR) of the nasopharyngeal swab tested positive for the SARS-CoV-2 nucleic acid, thereby confirming the diagnosis of COVID-19. He was hospitalized for 3 days. Antibiotic therapy (azithromycin) was administered.

Eleven days after hospital discharge (afebrile for more than 1 week), the patient was readmitted due to a 2-day history of fever, acute-onset right-sided back pain, and respiratory discomfort. His SpO_2_ was 93% on room air and >95% after oxygen supplementation (1L/min) by nasal catheter. On examination, he was afebrile, with a heart rate of 113 beats/min, blood pressure of 136/85 mmHg, and respiratory rate of 24 breaths/min. The thoracic auscultation indicated bilaterally reduced vesicular murmur with bibasilar crackles. Repeat non-enhanced chest CT showed an increase in the extent of bilateral and multilobar lung involvement by predominantly peripheral multifocal opacities, along with consolidation, and development of sub-pleural lines and newer ground-glass opacities (Figure 1B). A newly developed right-sided laminar pleural effusion was observed. An increase in the heart volume and pulmonary trunk diameter relative to the initial CT examination drew attention. The Wells score for PE was <2 points. The patient’s WBC count was 11090/mm^3^ (neutrophils, 8320/mm^3^; lymphocytes, 1600/mm^3^; monocytes, 1090/mm^3^), hemoglobin level was 13.9 g/dL, platelet count was 296000/mm^3^, serum D-dimer level was 2317 ng/mL Fibrinogen Equivalent Units (FEU; upper limit of normal, 500 ng/mL FEU), troponin I level was normal (<0.16 ng/mL), C-reaction protein level was 5.08 mg/dL (reference value, <1.00 mg/dL), lactate dehydrogenase level was 613 U/L (upper limit of normal, 480 U/L), prothrombin time was 14.2 s (reference range, 10.1-12.8 s), and activated partial thromboplastin time was 28.9 s (reference range, 25.4-33.4 s). A repeat rRT-PCR of nasopharyngeal swab tested positive for the SARS-CoV-2 nucleic acid. The patient underwent a transthoracic echocardiography that revealed normal findings. Since the right-sided back pain persisted on the second day of rehospitalization, CT pulmonary angiography was performed. It showed filling defects in sub-segmental arterial branches of the posterior basal segment of the right lower lobe, consistent with acute PE (Figure 1C and Figure 1D). Additionally, the peripheral lung opacity with reversed halo sign and central lucencies, compatible with pulmonary infarction, could be detected in this examination (Figure 1E). Dual-energy CT color-coded iodine “perfusion” map revealed only a focal iodine defect in the posterior basal segment of the right lower lobe, where the pulmonary infarction was detected (Figure 1F). The lupus anticoagulant testing was found positive. The patient was treated with a full-dose anticoagulant drug (subcutaneous enoxaparin) and antibiotics (azithromycin and piperacillin/tazobactam). He showed clinical and laboratory improvement, and was discharged in good general condition after 4 days of full anticoagulation, with rivaroxaban prescribed for home use.

## DISCUSSION

This case report described a patient infected with SARS-CoV-2 who developed acute PE several days after the onset of viral pneumonia. Before the outbreak of COVID-19, case reports had already described concurrent PE and viral lung infections^9^. Visseren et al.^10^concluded that respiratory viruses induce procoagulant activity in cultured human endothelial cells, although coronaviruses were not included in their study (published in 2000). A proinflammatory, hypercoagulable state may occur in patients with severe/critical COVID-19 symptoms, and possibly plays an important role in the development of PE^1^ ^,^ ^4^ ^,^ ^5^ ^,^ ^9^. Another factor that may favor the formation of PE is reduced physical movement due to quarantine restrictions or hospitalization (elevated risk of deep vein thrombosis)^4^. The published pathological reports and verbal communications have described presence of microvascular thrombosis and PE in the few cases with COVID-19 autopsied to date^4^. However, further studies are necessary to better comprehend the incidence of PE in the patients with COVID-19, and to ascertain the exact pathophysiological mechanisms involved.

Further, our case tested positive for lupus anticoagulant. Zhang et al.^11^ recently reported three COVID-19 cases with presence of thrombotic phenomena (multiple cerebral infarctions) and antiphospholipid antibodies. However, the cases were negative for lupus anticoagulant, but positive for anti-cardiolipin IgA and anti-*β*_2_-glycoprotein-I IgA and IgG antibodies.

Non-enhanced chest CT is considered to be the first-line imaging tool for evaluation of COVID-19 pneumonia^3^. Some ancillary findings on non-enhanced CT, such as peripheral lung opacities with features suggestive of pulmonary infarction (reversed halo sign/central lucencies), dilatation of pulmonary trunk, and increased cardiac volume (particularly, enlargement of right cardiac chamber) should indicate risk of PE^12^. These aspects, as observed in our patient, may aid in the decision to perform CT pulmonary angiography to confirm or exclude PE. Moreover, clinical parameters, including elevated D-dimer levels, hemoptysis, and sudden worsening of respiratory function or chest pain should also be considered^3^ ^,^ ^9^.Additionally, signs of right heart strain on electrocardiogram or echocardiogram may help in identifying patients with COVID-19 at risk of concurrent PE^9^. Whereas, contraindications associated with the use of intravenous iodinated contrast media, including allergy (prior allergy-like reaction to iodinated contrast medium) and renal dysfunction, should be weighed in the decision to perform CTPA^3^ ^,^ ^9^.

In conclusion, we should be aware of the association between COVID-19 and acute PE, and consider the clinical and laboratory findings as well as non-enhanced chest CT features (if performed) in the decision to perform a CTPA scan for patients infected with the novel coronavirus.
